# Z-DNA binding protein 1 mediates necroptotic and apoptotic cell death pathways in murine astrocytes following herpes simplex virus-1 infection

**DOI:** 10.1186/s12974-022-02469-z

**Published:** 2022-05-13

**Authors:** Austin M. Jeffries, Alexander J. Suptela, Ian Marriott

**Affiliations:** Department of Biological Sciences, University of North Carolina at Charlotte, 9201 University City Blvd., Charlotte, NC 28223 USA

**Keywords:** Astrocytes, Herpes simplex virus-1, ZBP1, Necroptosis, Apoptosis

## Abstract

**Background:**

The mechanisms by which glia respond to viral central nervous system (CNS) pathogens are now becoming apparent with the demonstration that microglia and astrocytes express an array of pattern recognition receptors that include intracellular RNA and DNA sensors. We have previously demonstrated that glia express Z-DNA binding protein 1 (ZBP1) and showed that this cytosolic nucleic acid sensor contributes to the inflammatory/neurotoxic responses of these cells to herpes simplex virus-1 (HSV-1). However, the relative contribution made by ZBP1- to HSV-1-mediated cell death in glia has not been determined.

**Methods:**

We have investigated the relative contribution made by ZBP1- to HSV-1-mediated cell death in primary astrocytes derived from mice genetically deficient in this sensor. We have used capture ELISAs and immunoblot analysis to assess inflammatory cytokine production and ZBP1 and phosphorylated mixed lineage kinase domain-like protein (MLKL) expression levels, respectively, following HSV-1 challenge. Furthermore, we have used a commercially available cell viability assay to determine the proportion and rate of cell death in cells following infection with laboratory and neuroinvasive clinical strains of HSV-1, and pharmacological inhibitors of necroptotic and apoptotic pathway components to assess the relative role of each.

**Results:**

We show that the loss of ZBP1 in astrocytes results in an increase in the number of viral particles released following HSV-1 infection. Importantly, we have confirmed that HSV-1 induces necroptosis in astrocytes and have established the ability of ZBP1 to mediate this cell death pathway. Interestingly, while ZBP1 is best known for its role in necroptotic signaling, our findings indicate that this sensor can also contribute to virally induced apoptosis in these glia.

**Conclusions:**

Our findings indicate that ZBP1 serves as a restriction factor for HSV-1 infection and is associated with the induction of both necroptotic and apoptotic cell death pathways in primary murine astrocytes. While it remains to be seen whether ZBP1-mediated activation of cell death in astrocytes contributes significantly to host protection or, rather, exacerbates HSV-1 encephalitis pathology, the identification of such a role in resident CNS cells may represent a novel target for therapeutic intervention to reduce HSV encephalitis-associated morbidity and mortality.

**Supplementary Information:**

The online version contains supplementary material available at 10.1186/s12974-022-02469-z.

## Introduction

It is now recognized that glial cells, such as astrocytes, play a critical role in the production of immune mediators that contribute to both protective host defense and disease pathology within the central nervous system (CNS) [1–10]. The mechanisms by which glia recognize and respond to CNS pathogens are now becoming apparent with the demonstration that astrocytes express a wide range of pattern recognition receptors (PRRs) capable of sensing pathogen- and damage-associated molecular patterns (PAMPs and DAMPs, respectively) [3, 11, 12]. Similar to peripheral host cells, activation of glial PRRs initiates signaling cascades that lead to the production of soluble pro-inflammatory and/or antiviral mediators. Whether such production and release act in a beneficial or detrimental manner in the CNS during infection is less well understood, and appears to be context dependent [13].

Of these PRRs, the expression of recently discovered cytosolic/nuclear RNA and DNA sensors, such as Z-DNA binding protein 1 (ZBP1; also known as DNA-dependent activator of interferon regulatory factors (DAI)), by glial cells is of particular interest as their ability to interact with nucleic acids in the intracellular environment suggests an important role in the detection of viral CNS pathogens, such as herpes simplex virus type 1 (HSV-1) [1, 4]. Furthermore, ZBP1 has been demonstrated to be both protective and damaging to the host, depending on the context of the challenge [14–16]. This molecule was first identified as a DNA sensor capable of inducing type one interferon (IFN) expression in response to cytosolic DNA or viral infection [17]. However, ZBP1 was subsequently found to initiate nuclear factor kappa B (NF-κB) activation and pro-inflammatory mediator production following dsDNA stimulation [17–19]. In agreement with these studies, we have demonstrated that ZBP1 can contribute to pro-inflammatory mediator production during HSV-1 infection in murine glia, and shown that these mediators cause neuronal cell death [1, 4].

Recently, ZBP1 has also been found to initiate cell death pathways, such as necroptosis in non-CNS cell types [20–27]. Upon binding to nucleic acids, ZBP1 directly interacts with receptor-interacting protein kinase 3 (RIPK3) via their RIP homotypic interaction motif (RHIM) domains [19, 27, 28]. Following this interaction, RIPK3 phosphorylates mixed lineage kinase domain-like protein (MLKL) to induce necroptosis [29]. While necroptosis can limit viral dissemination by killing the host cell prior to viral replication, it may also exacerbate damaging pro-inflammatory responses [30].

Here, we have investigated the contribution made by ZBP1- to HSV-1-mediated cell death in murine astrocytes. We confirm that HSV-1 induces necroptosis in this cell type and have established the ability of ZBP1 to initiate this cell death pathway in glia. Interestingly, while ZBP1 is best known for its role in necroptotic signaling, our findings indicate that this sensor can also contribute to virally induced apoptosis in astrocytes. Together, our findings suggest that ZBP1 serves as a restriction factor for DNA virus infection via the induction of cell death pathways in these non-neuronal CNS cells.

## Materials and methods

### Murine astrocyte isolation and culture

Primary murine astrocytes were isolated as described previously by our laboratory [1, 31–33] from neonatal C57BL/6 J (ZBP1 + / +) or ZBP1-deficient mice on a C57BL/6 J background (ZBP1−/−). ZBP1+/+ mice were purchased from Jackson Laboratories and ZBP1−/− animals generated by CRISPR-Cas9 technology [34] were a kind gift from Dr. Laura Knoll (University of Wisconsin-Madison, Madison, WI). Briefly, six to eight brains per preparation from mouse neonates were dissected free of meninges and large blood vessels and finely minced with sterile surgical scissors. The minced tissue was then forced through a wire screen and briefly incubated with 0.25% trypsin 1 mM EDTA in serum-free RPMI 1640 medium for 5 min. The cell suspension was then washed, and this mixed glial culture was maintained in RPMI 1640 containing 10% fetal bovine serum (FBS) and penicillin–streptomycin mix for two weeks.

Astrocytes were isolated from mixed glial cultures by trypsinization (0.25% trypsin-1 mM EDTA for 20–30 min) in the absence of FBS as previously described [1, 35], and were determined to be > 96% pure based on morphological characteristics and the expression of the astrocyte marker glial fibrillary acidic protein (GFAP) as determined by immunofluorescence microscopy [35]. These cells were cultured in RPMI 1640 containing 10% FBS. All studies were performed in accordance with relevant federal guidelines and institutional polices regarding the use of animals for research purposes.

### Preparation of viral stocks and in vitro infection of glial cells

HSV-1 viral stocks were prepared by infecting monolayer cultures of Vero cells (ATCC; CCL-81) with HSV-1 MacIntyre strain (HSV-1(MacIntyre)) from a patient with encephalitis (ATCC; VR-539), or an ICP6 RHIM mutant strain (HSV-1(F)-ICP6-RHIM Mut) or its parental F strain (HSV-1(F)) (kind gifts from Dr. Edward Mocarski of Emory University, Atlanta, GA) at a multiplicity of infection (MOI) of 0.01 and incubated for 48 to 72 h, at which time 100% of cells displayed cytopathic effects. Tissue culture flasks were then placed at − 80 °C for 15 min and subsequently warmed to room temperature inside a tissue culture hood. The cell suspension was removed and pulse sonicated (Vibra Cell; Sonics and Materials Inc., Newton, CT) to release intake virions. The sonicated material was centrifuged at 4000 RCF to remove unwanted cell debris and the supernatant mixed with sterile milk for increased stability during freeze/thaw cycles. The stock was aliquoted and viral titers were quantified using a standard plaque assay of serial dilutions on Vero cells at 37 °C. The viral titer of the stock solutions was 1.2 × 10^7^ PFU/ml for HSV-1(MacIntyre) and HSV-1(F)-ICP6-RHIM Mut, and 1.5 × 10^7^ PFU/ml for HSV-1(F). Murine astrocytes were infected with HSV-1 at MOIs of 0.02, 0.2, or 2.0 viral particles to glia, and the virus was allowed to adsorb for 1 h in DMEM in the absence of FBS or antibiotics. Cells were subsequently washed with PBS and cultures were maintained in appropriate growth medium for the indicated times prior to measuring cell viability or the collection of supernatants and/or whole-cell protein isolates. In experiments with inhibitors, following infection, the signal transducer and activator of transcription 1 (STAT1) inhibitor Fludarabine (10 μM; Selleckchem), RIPK1 inhibitors GSK963 and GSK547 (1 μM, 50 nM; Sigma, Selleckchem), RIPK3 inhibitors GSK872 and GSK843 (5 μM, 2 µM; Sigma), the pan caspase inhibitor Z-VAD-FMK (20 μM; InvivoGen), and the caspase-8 inhibitor Z-IETD-FMK (20 μM; InvivoGen) were reconstituted in DMSO and added to the cultures.

### Immunoblot analysis

Whole-cell protein isolates were collected from astrocytes using Triton lysis buffer (10 mM Tris HCl pH 10.5, 5 mM MgCl_2_, and 1% (v/v) Triton X-100) and analyzed by immunoblot analysis. Samples were electrophoresed on a 12% SDS–polyacrylamide gel and transferred to Immobilon-P transfer membranes (Millipore). Membranes were blocked with either 5% milk (for ZBP1) or 5% BSA (for P-MLKL) for 1 h and then incubated overnight at 4 °C with primary antibodies directed against ZBP1 (AdipoGen) or pMLKL (Abcam) and the housekeeping gene product β-actin (Abcam). Blots were then washed and incubated in the presence of a horseradish peroxidase (HRP)–conjugated anti-rabbit or anti-mouse IgG secondary antibody. Bound enzyme was detected with the Super Signal system (Thermo Fisher Scientific). Immunoblots shown are representative of at least three separate experiments using the Bio-Rad ChemiDoc imaging system, and quantification analysis was performed using ImageLab software (Bio-Rad).

### Enzyme-linked immunosorbent assay

Specific capture enzyme-linked immunosorbent assays (ELISAs) were performed to quantify murine IL-6, IFN-β, or TNF release. The murine IL-6 ELISA was conducted using a rat anti-mouse IL-6 capture antibody (BD Pharmingen) and a biotinylated rat anti-mouse IL-6 detection antibody (BD Pharmingen). The murine IFN-β ELISA was carried out using a polyclonal goat anti-mouse IFN-β capture antibody (Biolegend) and a biotinylated Armenian hamster anti-mouse IFN-β detection antibody (Biolegend). The murine TNF ELISA was conducted using a commercially available kit (R&D Systems DuoSet ELISA). Bound antibody was detected using streptavidin-HRP (BD Biosciences) followed by the addition of tetramethylbenzidine (TMB) substrate. H_2_SO_4_ was used to stop the reaction and absorbance was measured at 450 nm. Dilutions of murine IL-6, IFN-β, and TNF (BD Biosciences, Biolegend, R&D systems, respectively) were used to generate standard curves, and the concentration of each in study samples was determined by extrapolation to the standard curve.

### Measuring cell viability and calculation of the percentage and kinetics of cell death

Cell viability was measured for 24 h post-infection in isolated murine astrocytes using RealTime-Glo™ MT cell viability assay (Promega) according to the manufacturer’s instructions. Briefly, the NanoLuc® enzyme and MT cell viability substrate were combined with the appropriate growth media, with or without necroptosis or apoptosis pathway inhibitors, and added to glial cultures at one hour post-infection. Luciferase activity was measured every two hours using the SpectraMax® iD5 plate reader for 24 h beginning at two hours following infection. Luciferase readings were normalized to uninfected controls and the resulting values were subtracted from a value of one and multiplied by 100% to calculate percentage cell death. Data were further normalized within experimental groups by subtracting the percentage of dead cells at 2 h from values at all subsequent time points. Negative values were recorded as zero. The slopes and standard deviations for each treatment were determined by linear regression using Microsoft Excel software.

### Statistical analysis

Data are presented as the mean ± standard error of the mean (SEM). Statistical analyses were performed by one- or two-way analysis of variance (ANOVA) with Bonferroni’s or Tukey’s post hoc tests, or Student’s *t* test as appropriate using commercially available software (GraphPad Prism, GraphPad Software, La Jolla, CA). In all experiments, results were considered statistically significant when a p value of less than 0.05 was obtained.

## Results

### ZBP1 functions as an HSV-1 restriction factor in primary astrocytes

**Fig. 1:**
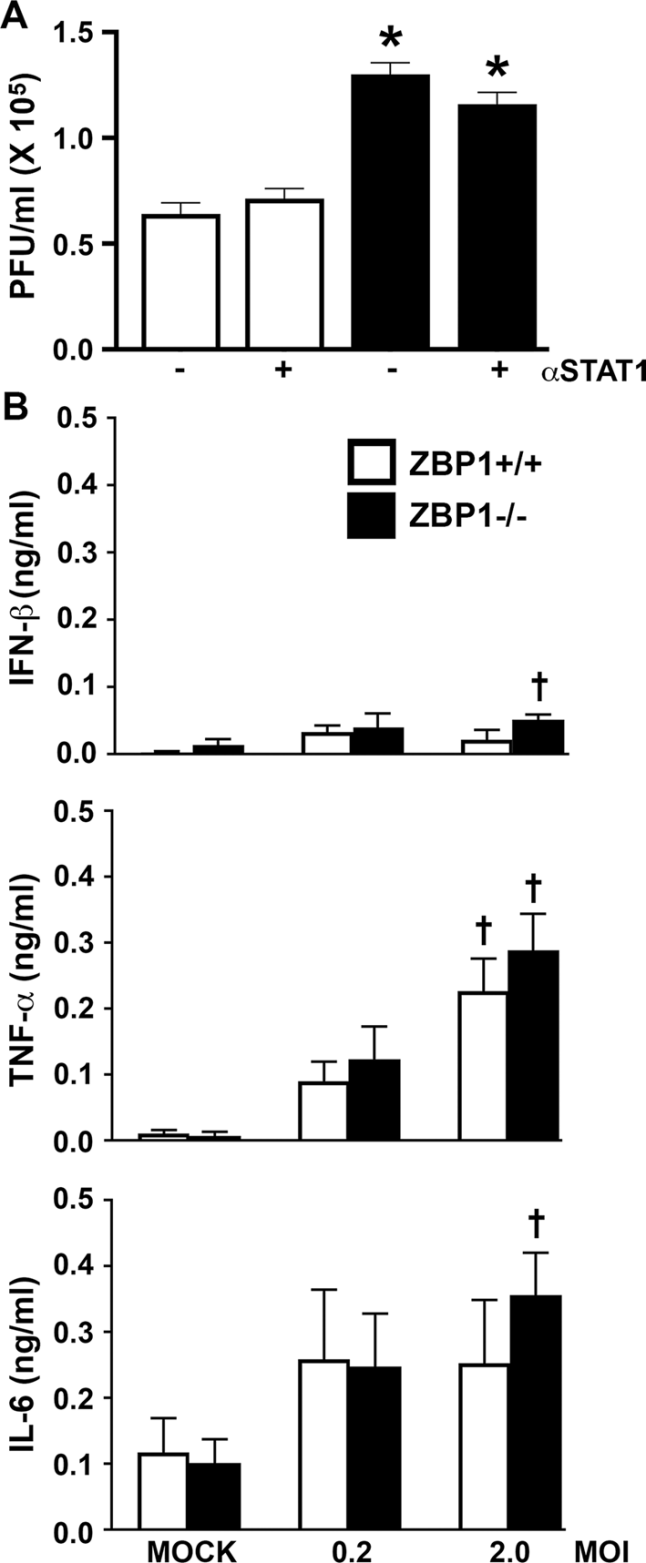
ZBP1 restricts HSV-1 replication in astrocytes in a manner that is independent of interferon production. **A** Murine astrocytes derived from wild-type mice (ZBP1 + / +) or animals deficient in the expression of ZBP1 (ZBP1−/−) were infected with HSV-1(MacIntyre) at an MOI of 2.0 for 60 min and then untreated or treated with the STAT1 inhibitor Fludarabine (10 μM). At 24 h, cell-free supernatants were collected and the number of plaque-forming units (PFU) released from HSV-1-infected astrocytes was determined by conventional plaque assays in Vero cells. **B** Murine astrocytes derived from ZBP1+/+ or ZBP1−/− mice were infected with HSV-1 at an MOI of 0.2 or 2.0. At 24 h, the concentration of IFN-β, IL-6, and TNF, in cell-free supernatants was quantified by specific capture ELISAs. Data are presented as the mean of at least three independent experimental replicates ± the SEM. An asterisk indicates a significant difference from similarly treated ZBP1+/+ cells and dagger symbols indicate a significant difference from mock infected cells (*p* < 0.05; *n* = 3)

ZBP1 has previously been demonstrated to act as an HSV-1 restriction factor in peripheral myeloid cells [20]. Here, we have investigated the ability of this sensor to limit infection in astrocytes derived from ZBP1+/+ and ZBP1−/− mice. Lack of ZBP1 protein expression was confirmed in astrocytes derived from ZBP1 knockout mice by immunoblot analysis (Additional file 1: Figure S1). ZBP1+/+ and ZBP1- astrocytes were infected with a clinical neuroinvasive HSV-1(MacIntyre) and the number of PFU released from infected cells was determined by conventional plaque assays in Vero cells. As shown in Fig. 1A, infectious viral particle release by ZBP1-deficient astrocytes was significantly greater than that seen with wild-type cells, although it should be noted that we have not directly assessed the amount of cell-associated virus in these studies.

To determine whether the higher levels of viral release were due to a reduction in the production of antiviral mediators, we measured IFN-β secretion in astrocytes following HSV-1 infection. As shown in Fig. 1B, both ZBP1+/+ and ZBP1−/− derived astrocytes produced only low levels of IFN-β production, with statistically significant amounts only being seen in ZBP1−/− derived astrocytes with HSV-1 at the higher MOI (2.0). Furthermore, we determined that treatment of either ZBP1+/+ or ZBP1−/− derived astrocytes with the STAT1 inhibitor, Fludarabine, had no effect on infectious particle release (Fig. 1A). In addition, we assessed the effect of genetic ZBP1 deficiency on HSV-1-induced production of the inflammatory cytokines IL-6 and TNF by these cells, and we report that ZBP1−/− deficient astrocytes release demonstrable levels of IL-6 and TNF following HSV-1 infection at the higher MOI (Fig. 1B). However, such production was not significantly different from that seen by ZBP1-expressing astrocytes following infection (Fig. 1B). Furthermore, the lower HSV-1 dose (0.2) failed to elicit significant production of any of these cytokines by either ZBP1+/+ or ZBP1−/− cells despite the significant difference seen in virus production at this MOI. As such, these data are inconsistent with ZBP1-mediated viral restriction being due to differences in cytokine production.

### HSV-1 infection induces necroptosis in astrocytes

**Fig. 2:**
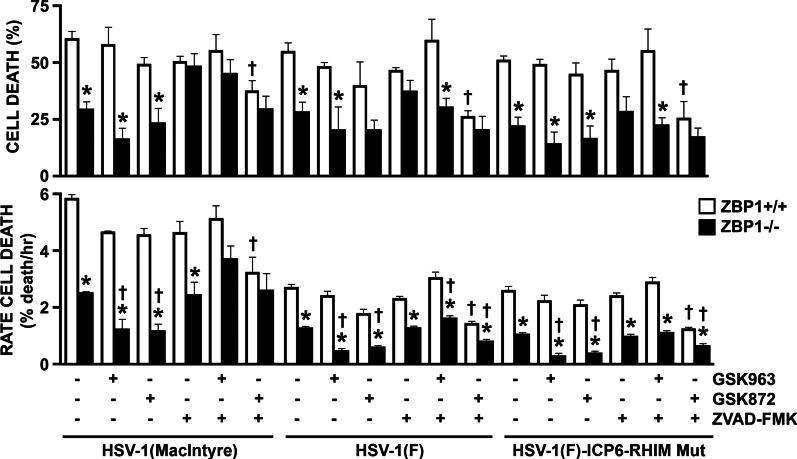
ZBP1 mediates HSV-1-induced cell death in primary murine astrocytes. ZBP1+/+ and ZBP1−/− murine astrocytes were infected with HSV-1(MacIntyre), HSV-1(F)-ICP6-RHIM Mut, or its parental ICP6-expressing parental strain (HSV-1(F)). One hour following infection, cells were treated with DMSO vehicle control, the RIPK1 inhibitor GSK963 (1 μM), the RIPK3 inhibitor GSK872 (5 μM), and/or the pan caspase inhibitor zVAD-FMK (20 μM). Cell viability was measured every two hours with a RealTime-Glo™ MT assay beginning at two hours following infection and data are reported as the percentage of dead cells at 24 h relative to non-infected controls (cell death) and as the rate of cell death. Data are shown as the mean of 3–6 independent experiments ± SEM. Asterisks indicate a significant difference from similarly treated ZBP1+/+ cells, while dagger symbols indicate significant difference from similarly challenged cells treated with DMSO vehicle only

**Fig. 3:**
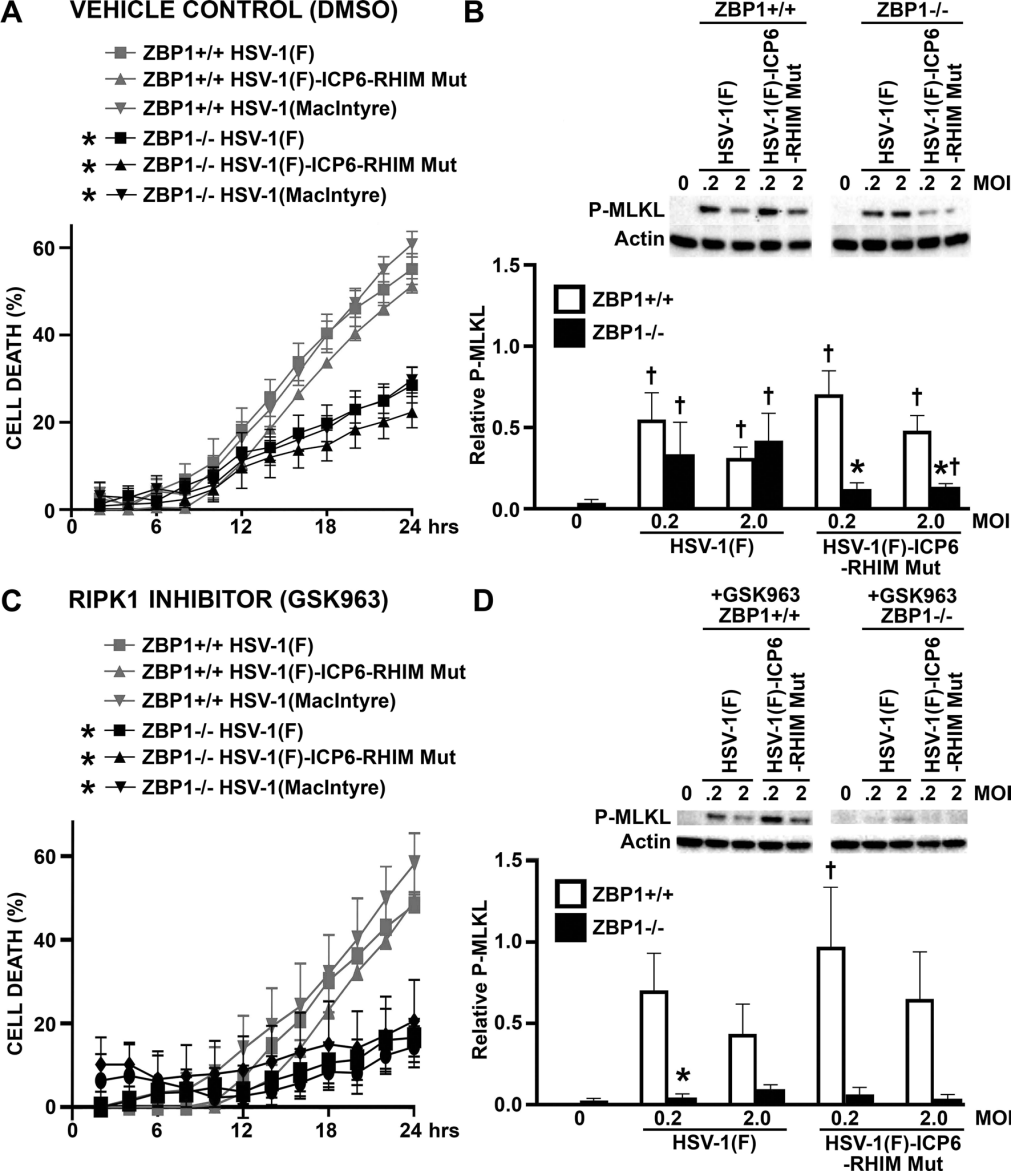
HSV-1 induces necroptotic cell death in primary astrocytes by both a RIPK1-independent ZBP1-mediated pathway and a RIPK1-mediated ZBP1-independent pathway. ZBP1+/+ and ZBP1−/− murine astrocytes were infected with HSV-1(MacIntyre), HSV-1(F)-ICP6-RHIM Mut, or its parental ICP6-expressing parental strain (HSV-1(F)). One hour following infection, cells were treated with either DMSO vehicle control (**A**, **B**) or the RIPK1 inhibitor GSK963 (1 μM) (**C**, **D**). **A**, **C** Cell viability was measured every two hours with a RealTime-Glo™ MT assay beginning at two hours following infection. **B**, **D** At 24 h, total cell lysates were collected and analyzed for the presence of phosphorylated MLKL (P-MLKL) or the housekeeping gene product β-actin (Actin) by immunoblot analysis. Relative P-MLKL expression was determined by densitometric analysis and normalized to β-actin expression levels. Data are shown as the mean of three independent experiments ± SEM. An asterisk indicates a significant difference in final cell death or P-MLKL expression from similarly treated ZBP1+/+ cells and dagger symbols indicate a significant difference from uninfected cells

Several studies have shown that ZBP1 can mediate necroptosis in non-CNS cell types [20–28]. To determine if this is also true in glia, we measured the rate of cell death in astrocytes derived from ZBP1+/+ and ZBP1−/− mice following HSV-1 infection. As shown in Figs. 2 and 3A, there was significant difference between ZBP1+/+ and ZBP1−/− derived astrocytes in the rate and final percentage of cell death at 24 h following challenge with HSV-1(MacIntyre).

However, it has recently been discovered that the HSV-1 gene product ICP-6 has a RHIM domain that is capable of directly interacting with receptor-interacting protein kinase 1 (RIPK1) and initiating necroptosis in mouse but not human cells [20, 36, 37]. To circumvent this possibility, and to more closely resemble responses in human cells, we have also assessed the ability of an HSV-1 strain with mutations in the ICP6 RHIM domain (HSV-1(F)-ICP6-RHIM Mut), and its parental F strain (HSV-1(F), to induce cell death in astrocytes in the presence and absence of ZBP1 expression. Interestingly, there was a significant difference between the rate and percentage of cell death at 24 h in ZBP1+/+ and ZBP1−/− derived astrocytes for both the HSV-1(F)-ICP6-RHIM Mut virus and the parental ICP6-expressing HSV-1(F) strain (Figs. 2 and 3A). While the decreased cell death induced by HSV-1(F)-ICP6-RHIM Mut in ZBP1−/− astrocytes correlates with a significant reduction in the level of phosphorylated MLKL (Fig. 3B), decreased cell death seen following infection with HSV-1(F) occurred despite similar levels of phosphorylated MLKL to those seen in ZBP1+/+ cells, suggesting that necroptosis is not solely responsible for HSV-1-induced cell death.

**Fig. 4:**
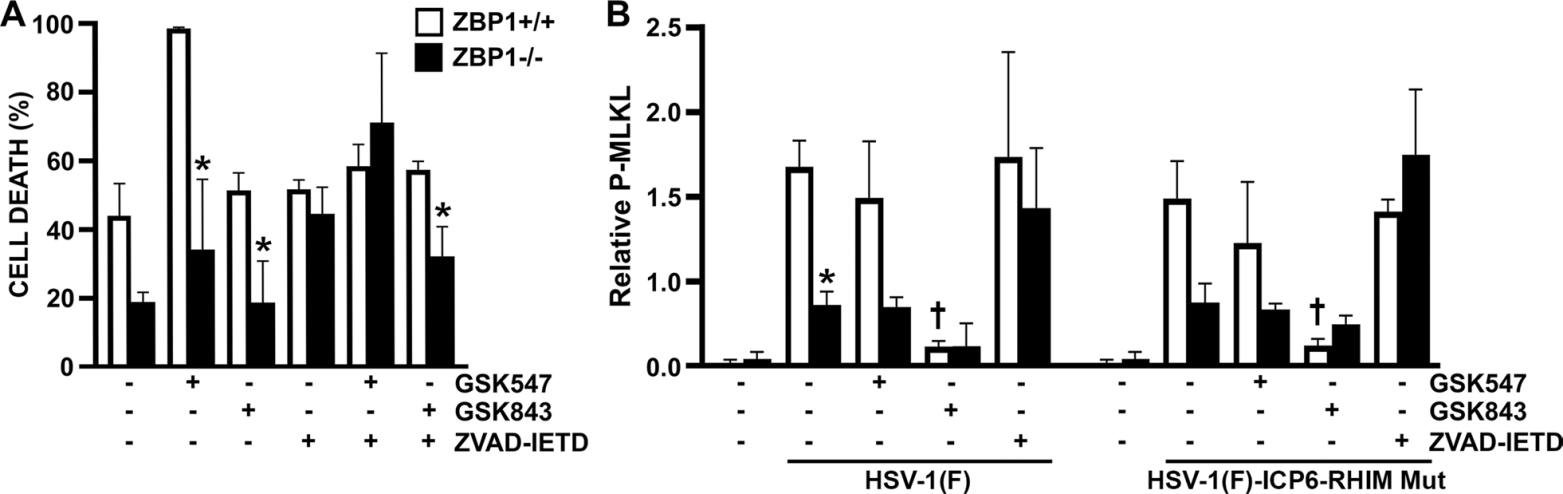
The notion that ZBP1 mediates multiple cell death pathways in HSV-1 challenged primary murine astrocytes is supported by similar results obtained using alternative pharmacological inhibitors. ZBP1+/+ and ZBP1−/− murine astrocytes were infected with HSV-1(MacIntyre) (**A**), or HSV-1(F)-ICP6-RHIM Mut or its parental ICP6-expressing parental strain (HSV-1(F)) (**B**). One hour following infection, cells were treated with either DMSO vehicle control, the RIPK1 inhibitor GSK547 (50 nM), the RIPK3 inhibitor GSK843 (2 μM), and/or the caspase-8 inhibitor Z-IETD-FMK (20 μM). **A** Cell viability was measured at 24 h following infection with a RealTime-Glo™ MT assay. Panel B: At 24 h, total cell lysates were collected and analyzed for the presence of phosphorylated MLKL (P-MLKL). Relative P-MLKL expression determined by densitometric analysis is shown normalized to β-actin expression levels. Data are shown as the mean of three independent experiments ± SEM. An asterisk indicates a significant difference in final cell death or P-MLKL expression from similarly treated ZBP1+/+ cells and dagger symbols indicate a significant difference from untreated infected cells

To determine if RIPK1 mediates MLKL phosphorylation in ZBP1-deficient astrocytes following infection, we treated ZBP1+/+ and ZBP1−/− derived astrocytes with the RIPK1 inhibitor, GSK963, during infection with the HSV-1(MacIntyre), HSV-1(F)-ICP6-RHIM Mut, and HSV-1(F) strains. The absence of a direct effect of GSK963, or other inhibitors used in this study, on cell death at 24 h and rate of cell death was confirmed in uninfected glial cells (Additional file 2: Figure S2). As shown in Figs. 2 and 3C, there remained a significant difference between ZBP1-expressing and ZBP1-deficient astrocytes in the rate and final percentage of cell death at 24 h in following challenge with HSV-1(MacIntyre) in the presence of GSK963. Similar results were obtained when another inhibitor of RIPK1, GSK547, was employed (Fig. 4A). Furthermore, there was a significant difference between the rate and percentage of cell death at 24 h in ZBP1+/+ and ZBP1−/− derived astrocytes for both the HSV-1(F)-ICP6-RHIM Mut and HSV-1(F) strains (Figs. 2 and 3C) following GSK963 treatment. Together, these data indicate that ZBP1-dependent differences in virally induced astrocytes cell death are not mediated by RIPK1 kinase activity.

Interestingly, treatment of ZBP1−/− derived astrocytes with GSK963 significantly reduced levels of phosphorylated MLKL induced by HSV-1(F) (Fig. 3D), with similar trends seen in experiments using GSK547 (Fig. 4B), suggesting that the MLKL phosphorylation seen in the absence of ZBP1 (Fig. 3B) is dependent on RIPK1 activity. Together, these data indicate that the HSV-1 is capable of inducing necroptosis in primary astrocytes by both a RIPK1-independent ZBP1-mediated pathway and a RIPK1-mediated ZBP1-independent pathway.

### ZBP1 mediates both necroptotic and apoptotic death pathways in virally challenged astrocytes

**Fig. 5:**
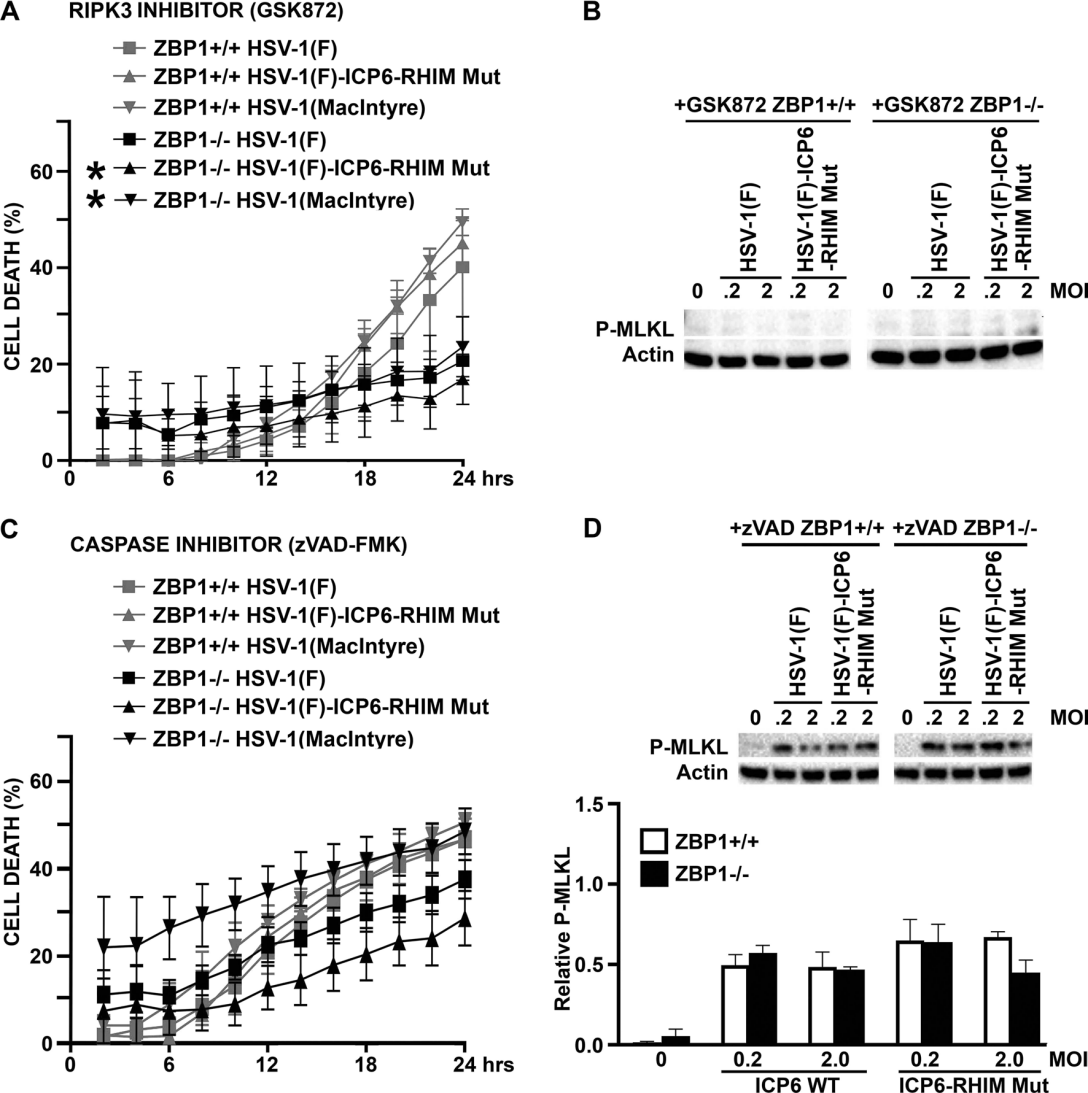
ZBP1-mediated cell death does not exclusively occur in HSV-1-infected astrocytes via necroptosis. ZBP1+/+ or ZBP1−/− murine astrocytes were infected with HSV-1(MacIntyre), HSV-1(F)-ICP6-RHIM Mut, or its parental ICP6-expressing parental strain (HSV-1(F)). One hour following infection, cells were treated with either the RIPK3 inhibitor GSK872 (5 μM) (**A**, **B**) or the pan caspase inhibitor zVAD-FMK (20 μM) (**C**, **D**). **A**, **C** Cell viability was measured every two hours with a RealTime-Glo™ MT assay beginning at two hours following infection. **B**, **D** At 24 h, total cell lysates were collected and analyzed for the presence of phosphorylated MLKL (P-MLKL) or the housekeeping gene product β-actin (Actin) by immunoblot analysis. Relative P-MLKL expression was determined by densitometric analysis and normalized to β-actin expression levels. Data are shown as the mean of three independent experiments ± SEM. Asterisks indicate a significant difference in final cell death from similarly treated ZBP1+/+ cells

Since both RIPK1- and ZBP1-mediated necroptosis have been shown to require RIPK3 activity to phosphorylate MLKL [21, 29], we have inhibited RIPK3 with the inhibitor GSK872 to determine whether necroptosis is the primary mechanism underlying HSV-1-induced cell death. Surprisingly, a significant difference remained between ZBP1-expressing and ZBP1-deficient astrocytes in the rate of death following challenge with the neuroinvasive HSV-1(MacIntyre) clinical HSV-1 strain, the HSV-1(F)-ICP6-RHIM Mut virus, or the parental HSV-1(F) ICP6-expressing strain, and in the final percentage of cell death at 24 h for the HSV-1(MacIntyre) and HSV-1(F)-ICP6-RHIM Mut strains (Figs. 2 and 5A) following treatment with the RIPK3 inhibitor, despite the absence of detectable phosphorylated MLKL expression (Fig. 5B). Similar results were obtained when another inhibitor of RIPK3, GSK843, was employed, with a significant difference remaining between ZBP1-expressing and ZBP1-deficient astrocytes in the final percentage of cell death at 24 h following challenge with HSV-1(MacIntyre) (Fig. 4A) or HSV-1(F) (data not shown), despite very low levels of phosphorylated MLKL (Fig. 4B). As such, these data indicate that necroptosis is not the sole mechanism underlying ZBP1-mediated astrocytic cell death.

To determine whether ZBP1-mediated astrocyte cell death also occurs via apoptosis, we performed parallel experiments using the pan caspase inhibitor zVAD-FMK or the caspase-8 inhibitor Z-IETD-FMK. As shown in Figs. 2 and 5C, pan caspase inhibition prevented significant differences in the final percentage of cell death at 24 h between ZBP1+/+ and ZBP1−/− derived cells following infection with any HSV-1 strain. Similarly, caspase-8 inhibition also prevented significant differences in the final percentage of cell death at 24 h between ZBP1+/+ and ZBP1−/− derived cells following infection with HSV-1(MacIntyre) (Fig. 4A) or HSV-1(F) (data not shown). In contrast, significant differences in the rates of cell death remained between cells expressing ZBP1 and those deficient in its expression following challenge with HSV-1(F) and HSV-1(F)-ICP6-RHIM Mut strains in the presence of the pan caspase inhibitor, but it is noteworthy that the lack of difference in final death percentage appears to be due primarily to a net increase in cell death in ZBP1−/− derived cells rather than a reduction in ZBP1+/+ cells (Figs. 2, 4A, and 5C).

Some studies have suggested that caspase inhibition may promote RIPK1 activation leading to necroptosis [38]. To assess this possibility, we measured phosphorylated MLKL protein levels in HSV-1(F) and HSV-1(F)-ICP6-RHIM Mut-infected astrocytes following caspase-8 or pan caspase inhibition. As shown in Figs. 4B and 5D, both ZBP1+/+ and ZBP1−/− derived astrocytes showed similar levels of phosphorylated MLKL with either HSV-1 strain in the presence of Z-IETD-FMK or zVAD-FMK, respectively. These results demonstrate that ZBP1-independent necroptosis in astrocytes occurs in the absence of caspase activity.

**Fig. 6:**
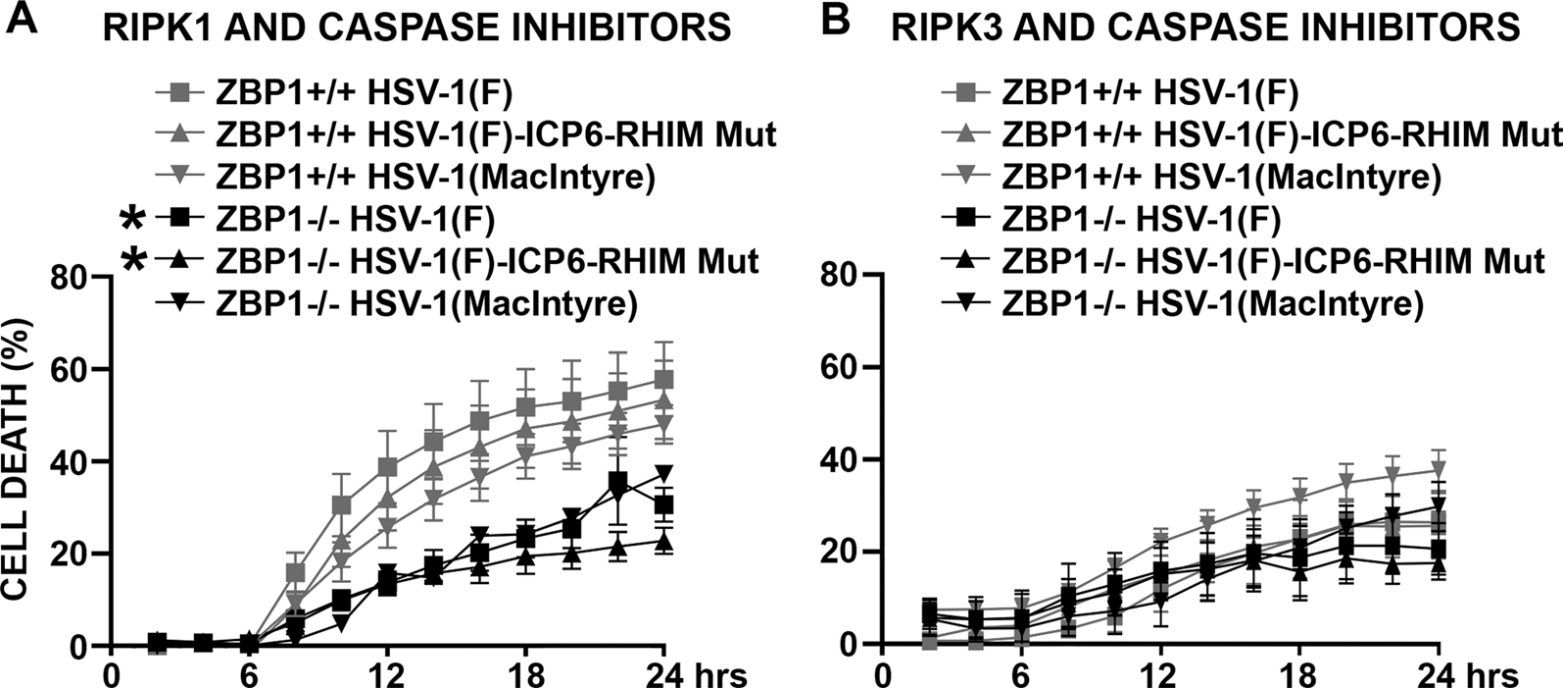
ZBP1 mediates both apoptotic and necroptotic cell death pathways in HSV-1 challenged primary murine astrocytes. ZBP1+/+ or ZBP1−/− derived astrocytes were infected with HSV-1(MacIntyre), HSV-1(F)-ICP6-RHIM Mut, or its parental ICP6-expressing parental strain (HSV-1(F)). One hour following infection, cells were treated with either the RIPK1 inhibitor GSK963 (1 μM) plus the pan caspase inhibitor zVAD-FMK (20 μM) (**A**) or the RIPK3 inhibitor GSK872 (5 μM) plus zVAD-FMK (20 μM) (**B**). Cell viability was measured every two hours with a RealTime-Glo™ MT assay beginning at two hours following infection. Data are shown as the mean of 4–6 independent experiments ± SEM. Asterisks indicate a significant difference in final cell death from similarly treated ZBP1+/+ cells

To directly determine if RIPK1 activation is responsible for the higher cell death rate seen in ZBP1-/ derived astrocytes following caspase inhibition, we simultaneously treated ZBP1+/+ or ZBP1−/− derived astrocytes with a RIPK1 inhibitor (GSK936) and a pan caspase inhibitor (zVAD-FMK) following infection with HSV-1(MacIntyre), HSV-1(F), and HSV-1(F)-ICP6-RHIM Mut strains. The percentage of cell death at 24 h (Figs. 2 and 6A) and kinetics of cell death remained significantly different between ZBP1+/+ and ZBP1−/− derived astrocytes in the presence of GSK936 and zVAD-FMK following challenge with either HSV-1(F) or HSV-1(F)-ICP6-RHIM Mut, indicating that caspase inhibition permits RIPK1-mediated necroptosis following infection. Similarly, the percentage of cell death at 24 h remained significantly different between ZBP1+/+ and ZBP1−/− derived astrocytes following challenge with HSV-1(F)-ICP6-RHIM Mut in the presence of the alternate RIPK1 inhibitor GSK547 and a caspase-8 inhibitor, with a similar trend seen with the HSV-1(F) strain (data not shown). However, it should be noted that neither the GSK936 and zVAD-FMK (Figs. 2 and 6A) nor the GSK547 and Z-IETD-FMK (Fig. 4A) inhibitor combinations prevented significant differences in the final percentage of cell death or kinetics of cell death between ZBP1+/+ and ZBP1−/− derived astrocytes challenged with HSV-1(MacIntyre).

Since neither pan caspase nor RIPK3 inhibition alone significantly reduced the percentage of cell death at 24 h in virally challenged ZBP+/+ derived astrocytes (Figs. 2 and 5), we investigated whether simultaneous activation of both pathways could account for the differences in cell death seen between ZBP1+/+ and ZBP−/− astrocytes. To accomplish this, we treated ZBP1+/+ and ZBP1−/− derived astrocytes with both a RIPK3 inhibitor (GSK872) and a pan caspase inhibitor (zVAD-FMK) following infection with HSV-1(MacIntyre), HSV-1(F), and HSV-1(F)-ICP6-RHIM Mut strains. The percentage cell death at 24 h and the death rates following infection of ZBP1+/+ cells with all strains were all significantly lower in the presence of this inhibitor combination (Figs. 2 and 6B). Interestingly, while the kinetics of cell death remained significantly different between ZBP1+/+ and ZBP1−/− derived cells following infection with the HSV-1(F) and HSV-1(F)-ICP6-RHIM Mut strains, the percentage cell death induced by all HSV-1 strains at 24 h was reduced in ZBP1+/+ derived astrocytes to levels that were not significantly different from those seen in ZBP1−/− astrocytes (Figs. 2 and 6), and co-treatment significantly increased PFU release from wild-type ZBP1-expressing astrocytes as assessed by plaque assays in Vero cells (13.93 ± 0.52 X 10^5^ PFU/ml versus 0.64 ± 0.06 X 10^5^ PFU/ml in GSK872/ zVAD-FMK treated versus untreated cells, respectively, p < 0.05, n = 3). However, it should be noted that this was not the case for HSV-1(MacIntyre)-infected cells treated with GSK843 and Z-IETD-FMK (Fig. 4B) and the reason for this disparity is unclear. Taken overall, however, these studies indicate that ZBP1 mediates both apoptotic and necroptotic cell death pathways in virally challenged primary murine astrocytes that can serve to restrict DNA virus replication.

## Discussion

HSV-1 is a highly successful neurotropic DNA virus and the most common cause of fatal sporadic encephalitis worldwide [39]. Damage caused during HSV encephalitis is attributable either to a lack of control of HSV-1 replication or the over-production of inflammatory mediators [40]. As such, it is critical to determine the mechanisms leading to the initiation of early immune responses and their relative contribution to protection or pathophysiology.

We have previously determined that murine glia express ZBP1 and showed that this cytosolic nucleic acid sensor contributes to glial inflammatory responses to HSV-1 that are damaging to neuronal cells [1, 4]. In the present study, we demonstrate a role for this sensor in HSV-1 restriction and the initiation of cell death pathways in glia. We show that the loss of ZBP1 in primary murine astrocytes results in a significant increase in the release of PFUs following infection with a neuroinvasive clinical strain of HSV-1 [41]. Interestingly, this increase in infectious viral particle release does not appear to result from changes in IFN or inflammatory cytokine production as evidenced by the minimal production of IFN-β by astrocytes following infection, the lack of effect of STAT1 inhibition on PFU increases, and the lack of a significant effect of ZBP1 deficiency on HSV-1-induced release of this antiviral mediator or the key inflammatory cytokines IL-6 and TNF.

While the present results might seem at odds with our prior studies utilizing siRNA approaches [4], it must be noted that we previously showed that infection of astrocytes with HSV-1(McIntyre) at a high dose (MOI of 10) elicits robust IL-6 and TNF production that is sensitive to siRNA-mediated ZBP1 knockdown, while lower MOIs do not. As such, the present demonstration that infection of murine astrocytes with HSV-1 at lower doses (MOI of 0.2–2.0) elicits low level inflammatory cytokine release that is not significantly different between wild-type and ZBP1-deficient cells is consistent with our previous findings.

Recently, ZBP1 has been identified as an important mediator of necroptosis during infection with DNA and RNA viruses [20, 21, 23, 25]. In agreement with these studies, we have demonstrated that HSV-1 can initiate necroptosis in a ZBP1-dependent manner in astrocytes, as evidenced by the activation of the necroptotic marker MLKL. Additionally, our results are also consistent with an ability of the HSV-1 product ICP6 to activate necroptosis in murine astrocytes independent of ZBP1 via RIPK1, an ability that has previously been described in other mouse cell types [20, 36, 37]. The detection of ICP6 and subsequent initiation of necroptosis could serve to restrict viral replication and/or exacerbate inflammatory host responses in mice. However, this HSV-1 product does not appear to induce necroptosis in human cells, and so we have additionally employed the HSV-1(F)-ICP6-RHIM Mut strain to circumvent direct ICP6-induced necroptosis in the present study to distinguish ZBP1-mediated effects that might be relevant to human cells.

Surprisingly, inhibition of necroptosis using RIPK3 inhibitors failed to significantly reduce the rate or percentage of cell death in ZBP1-expressing astrocytes to the levels seen in ZBP1-deficient cells following challenge with laboratory and clinical strains of HSV-1. Furthermore, inhibition of apoptosis with a pan caspase or caspase-8 inhibitors also failed to reduce virally induced cell death in ZBP1+/+ astrocytes. However, while inhibition of either pathway alone could not reduce cell death in WT astrocytes during infection, it was reduced by simultaneous inhibition of both apoptosis and necroptosis, and this was associated with a significant increase in infectious particle release as assessed by conventional plaque assays. This suggests that ZBP1 contributes to both cell death pathways in astrocytes and these serve to restrict replication of this DNA virus.

Interestingly, inhibition of apoptotic pathways appears to permit HSV-induced necroptosis in astrocytes that is independent of ZBP1, as treatment with a pan caspase or a caspase-8 inhibitor did not reduce levels of phosphorylated MLKL following infection in ZBP1-deficient cells. Indeed, inhibition of caspase-8 led to a seemingly paradoxical increase in the rate of death in ZBP1−/− derived astrocytes. However, prior studies have indicated that caspase-8 can act as a negative regulator of RIPK1-mediated necroptosis [42–45]. Furthermore, Mandal and coworkers [46] have previously demonstrated that RIPK3 can initiate apoptosis independent of its kinase activity. In these studies, they showed that RIPK3 can associate with RIPK1 via their respective RHIM domains in the presence of RIPK3 kinase inhibitors, leading to the recruitment and activation of caspase-8 that results in apoptosis [46]. In the present study, we have shown that increases in the rate of virally induced death in ZBP1−/− derived astrocytes following pan caspase inhibition are abolished by the simultaneous inhibition of RIPK1, consistent with a similar role for caspases as negative regulators of RIPK1-induced necroptosis in glia.

**Fig. 7:**
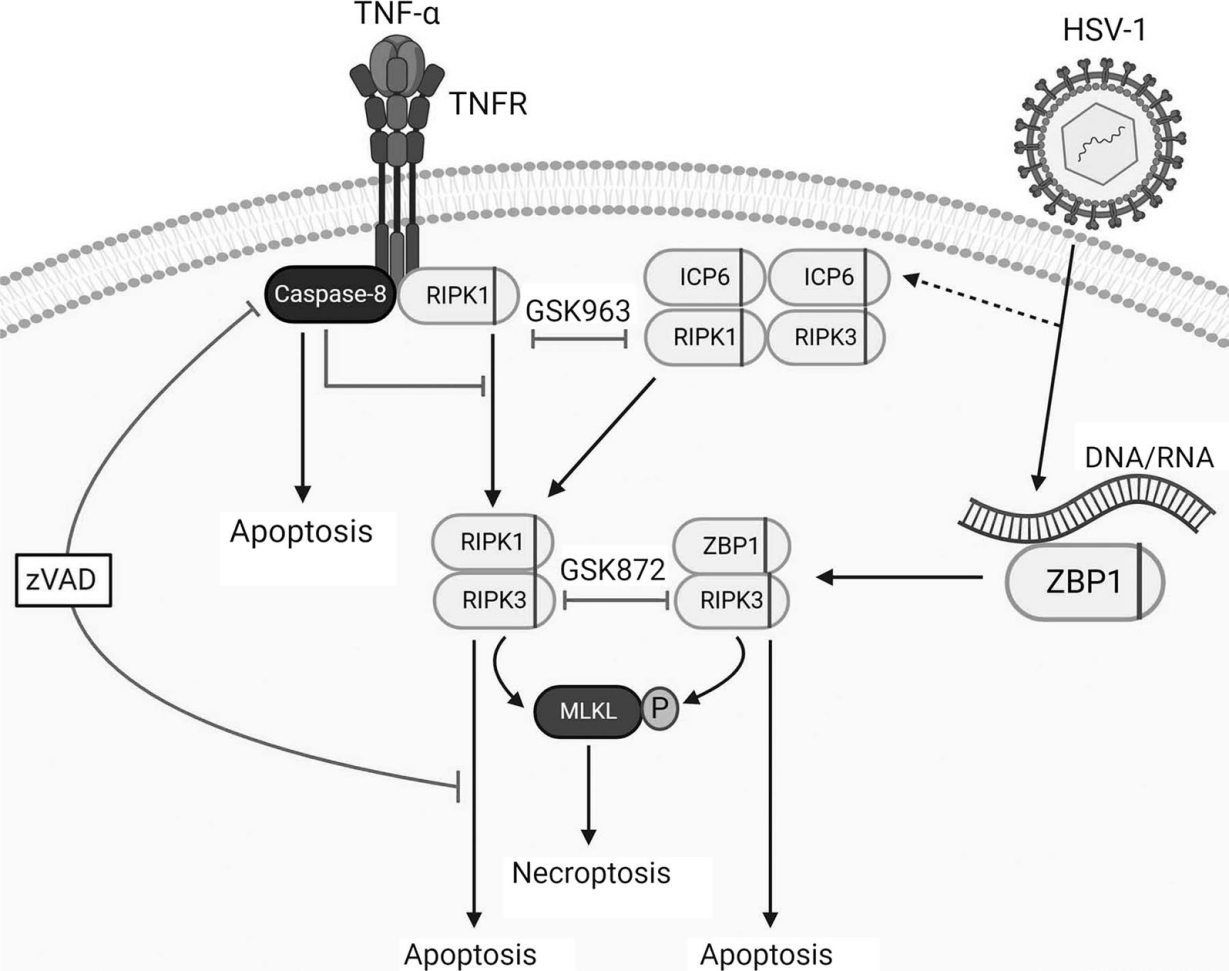
Proposed mechanisms underlying the activation of cell death pathways in murine astrocytes following HSV-1 challenge. The release of genetic material or transcription of HSV-1 genes leads to nucleic acid recognition by ZBP1 and its subsequent association with RIPK3 via their respective RHIM domains, which results in phosphorylation of MLKL and the execution of necroptosis. In addition, ZBP1 association with RIPK3 can induce apoptosis via an, as yet, unknown mechanism. Alternatively, the HSV-1 viral protein ICP6 can directly interact with RIPK1 and/or RIPK3 via each of their RHIM domains causing RIPK1 and RIPK3 to associate, thereby initiating necroptosis and apoptosis in a similar manner to that caused by ZBP1/RIPK3. Finally, viral infection elicits the production of TNF-α that can act in an autocrine/paracrine manner to initiate apoptosis in the presence of function caspase-8, or necroptosis via the association of RIPK1 with RIPK3 in the absence of functional caspase-8

As such, we propose that ZBP1 functions as an intracellular sensor for DNA viruses, such as HSV-1, and induces both apoptotic and necroptotic cell death signaling pathways in mouse astrocytes via multiple signaling pathways, as summarized in Fig. 6. In this model, the release of genetic material or transcription of HSV-1 genes leads to nucleic acid recognition by ZBP1 and its subsequent association with RIPK3 via their respective RHIM domains, which results in phosphorylation of MLKL and the execution of necroptosis. In addition, ZBP1 association with RIPK3 can also induce apoptosis via an, as yet, unknown mechanism. Alternatively, the HSV-1 viral protein ICP6 can directly interact with RIPK1 and/or RIPK3 via each of their RHIM domains causing RIPK1 and RIPK3 to associate, thereby initiating necroptosis and apoptosis in a similar manner to that caused by ZBP1/RIPK3. Lastly, viral infection elicits the production of TNF that can act in an autocrine/paracrine manner to initiate apoptosis in the presence of functional caspase-8, or necroptosis via the association of RIPK1 with RIPK3 in its absence (Fig. 7).

However, it should be noted that the present study does not preclude the involvement of other nucleic sensors, such as cGAS-STING, or even RIG-I, in glial immune and cell death responses to HSV-1, perhaps in a cooperative or cell-type specific manner. Indeed, a recent study has suggested that cGAS-STING can mediate type I IFN-independent apoptotic cell death in murine microglia in vitro and in vivo following HSV-1 infection at high doses [47]. Although, our studies suggest that this DNA sensor system fails to mediate effective antiviral immune responses to HSV-1 in human microglial cells, despite being important in their IFN-β responses (48). Furthermore, the relative importance of such ZBP1-mediated responses in host defense against DNA virus challenge remains to be determined in vivo and it is, as yet, unclear whether such mechanisms operate in human glia in response to HSV-1 infection.

## Conclusions

Taken in concert, we have shown that the cytosolic nucleic acid sensor ZBP1 serves as a restriction factor for HSV-1 in primary murine astrocytes and demonstrated that reductions in infectious viral particle release are associated with the induction of both necroptotic and apoptotic cell death pathways in infected cells. While it remains to be seen whether ZBP1-mediated activation of cell death contributes significantly to host protection or, rather, exacerbates DNA virus-associated pathology, the identification of such a role in resident CNS cells may represent a novel target for therapeutic intervention to reduce HSV encephalitis-associated morbidity and mortality.

## Supplementary Information

**Additional file 1: Figure S1.** Confirmation of ZBP1 deficiency in ZBP1−/− derived astrocytes. ZBP1+/+ and ZBP1−/− derived primary murine astrocytes were infected with HSV-1(F) or HSV-1(F)-ICP6-RHIM Mut and, one hour following infection, were treated with (A) DMSO (vehicle), (B) GSK963 (1 μM), (C) GSK872 (5 μM), (D) zVAD-FMK (20 μM). At 24 h following treatment total cell lysates were collected and analyzed for the presence of ZBP1 or the house keeping gene β-actin by immunoblot analysis.

**Additional file 2: Figure S2.** Treatment of uninfected primary murine astrocytes with DMSO, GSK963 (1 μM), GSK872 (5 μM), and zVAD-FMK (20 μM), alone or in combination, for up to 24 h, fails to elicit demonstrable effects on cell viability/proliferation as assessed by total luminescence after 24-h treatment or rate of change of signal in the RealTime-Glo™ cell viability assay.

## Data Availability

The data used and/or analyzed during the current study are available from the corresponding author on reasonable request.

## Abbreviations

ATCC: American type culture collection
ANOVA: Analysis of variance
BSA: Bovine serum albumin
CNS: Central nervous system
CRISPR: Clustered regularly interspaced short palindromic repeats
DAMP: Damage-associated molecular pattern
DMSO: Dimethyl sulfoxide
DMEM: Dulbecco’s modified Eagle’s medium
ELISA: Enzyme-linked immunosorbent assay
EDTA: Ethylenediaminetetraacetic acid
FBS: Fetal bovine serum
HSV-1: Herpes simplex virus-1
HRP: Horseradish peroxidase
IFN: Interferon
IL: Interleukin
MLKL: Mixed lineage kinase domain-like
MOI: Multiplicities of infection
PAMP: Pathogen-associated molecular pattern
PRR: Pattern recognition receptor
PBS: Phosphate-buffered saline
RHIM: Receptor-interacting protein homotypic interaction motif
RIPK1: Receptor-interacting protein kinase 1
RIPK3: Receptor-interacting protein kinase 3
STAT1: Signal transducer and activator of transcription 1
siRNA: Small interfering RNA
SDS: Sodium dodecyl sulfate
SEM: Standard error of the mean
TNF: Tumor necrosis factor
ZBP1: Z-DNA binding protein 1
