# Supplementary figures and images for: Z-DNA binding protein 1 mediates necroptotic and apoptotic cell death pathways in murine astrocytes following herpes simplex virus-1 infection
Source: J Neuroinflammation. 2022 May 13;19:109. doi: 10.1186/s12974-022-02469-z (PMC9103380; doi:10.1186/s12974-022-02469-z)

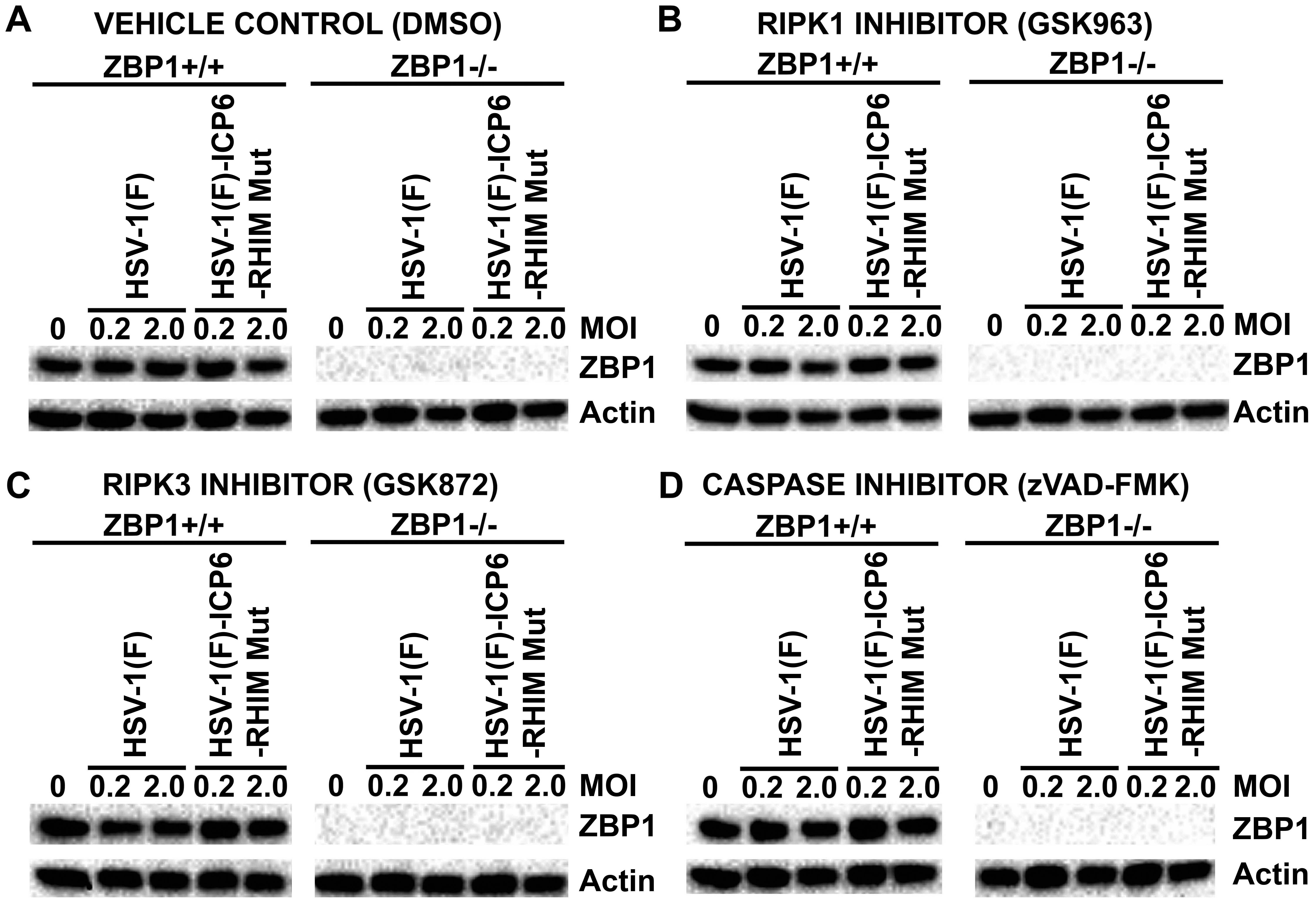

Supplement: Supplementary file 1 — Additional file 1: Figure S1. Confirmation of ZBP1 deficiency in ZBP1−/− derived astrocytes. ZBP1+/+ and ZBP1−/− derived primary murine astrocytes were infected with HSV-1(F) or HSV-1(F)-ICP6-RHIM Mut and, one hour following infection, were treated with (A) DMSO (vehicle), (B) GSK963 (1 μM), (C) GSK872 (5 μM), (D) zVAD-FMK (20 μM). At 24 h following treatment total cell lysates were collected and analyzed for the presence of ZBP1 or the house keeping gene β-actin by immunoblot analysis. [file 12974_2022_2469_MOESM1_ESM.jpg]

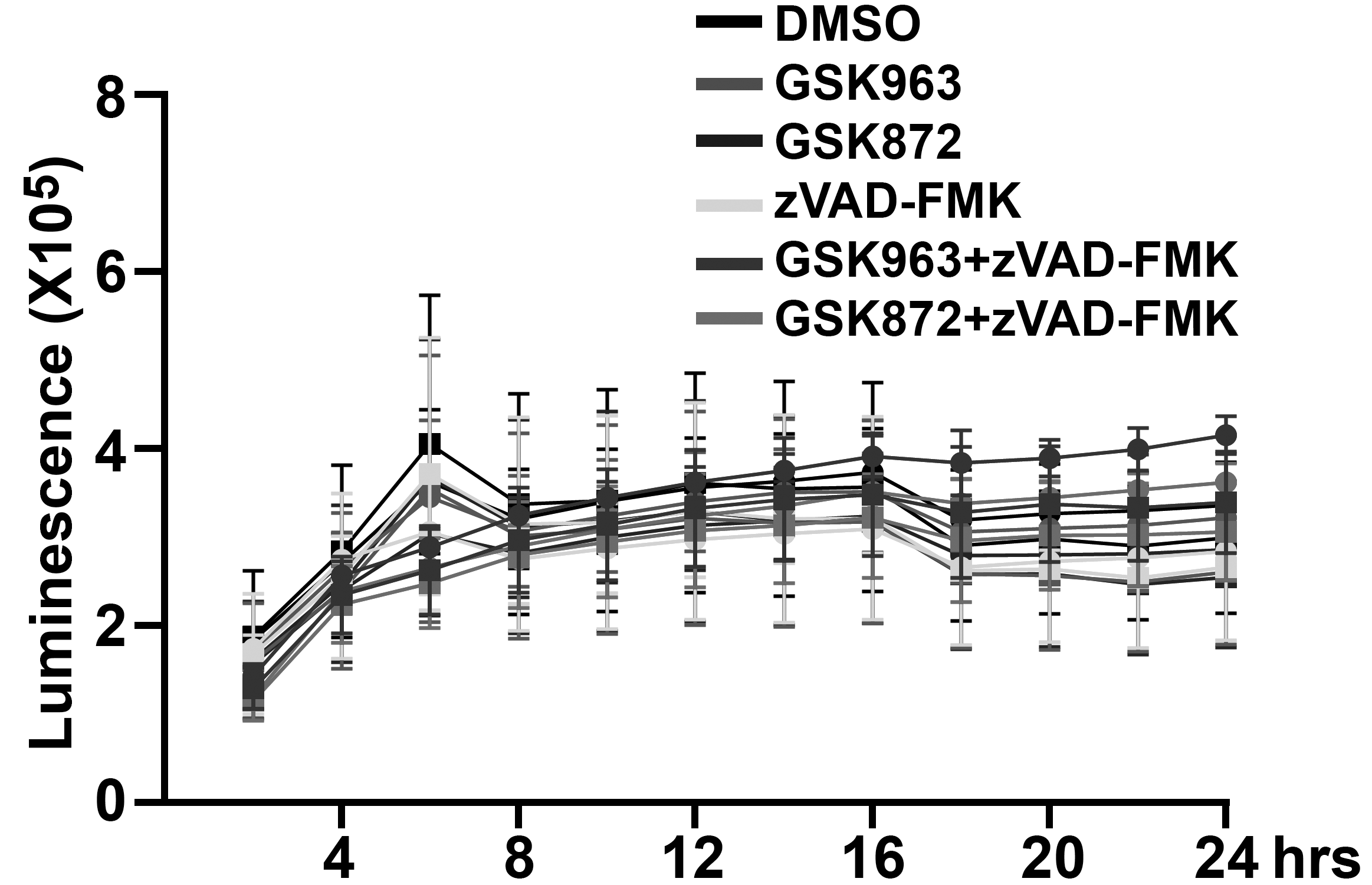

Supplement: Supplementary file 2 — Additional file 2: Figure S2. Treatment of uninfected primary murine astrocytes with DMSO, GSK963 (1 μM), GSK872 (5 μM), and zVAD-FMK (20 μM), alone or in combination, for up to 24 h, fails to elicit demonstrable effects on cell viability/proliferation as assessed by total luminescence after 24-h treatment or rate of change of signal in the RealTime-Glo™ cell viability assay. [file 12974_2022_2469_MOESM2_ESM.tif]
